# Feasibility Study of a Hand Guided Robotic Drill for Cochleostomy

**DOI:** 10.1155/2014/656325

**Published:** 2014-07-07

**Authors:** Peter Brett, Xinli Du, Masoud Zoka-Assadi, Chris Coulson, Andrew Reid, David Proops

**Affiliations:** ^1^Brunel Institute for Bioengineering, Brunel University, London UB8 3PH, UK; ^2^ENT Department, Queen Elizabeth Hospital, Birmingham B15 2TH, UK

## Abstract

The concept of a hand guided robotic drill has been inspired by an automated, arm supported robotic drill recently applied in clinical practice to produce cochleostomies without penetrating the endosteum ready for inserting cochlear electrodes. The smart tactile sensing scheme within the drill enables precise control of the state of interaction between tissues and tools in real-time. This paper reports development studies of the hand guided robotic drill where the same consistent outcomes, augmentation of surgeon control and skill, and similar reduction of induced disturbances on the hearing organ are achieved. The device operates with differing presentation of tissues resulting from variation in anatomy and demonstrates the ability to control or avoid penetration of tissue layers as required and to respond to intended rather than involuntary motion of the surgeon operator. The advantage of hand guided over an arm supported system is that it offers flexibility in adjusting the drilling trajectory. This can be important to initiate cutting on a hard convex tissue surface without slipping and then to proceed on the desired trajectory after cutting has commenced. The results for trials on phantoms show that drill unit compliance is an important factor in the design.

## 1. Introduction

Drilling through bone is a common operative task in surgical disciplines (ENT, neurosurgery, maxillofacial surgery, and orthopaedics are some examples). Surgeons within these fields are faced with the same challenges of cutting without slipping on hard bone surfaces, particularly with convex surfaces [1], and in discriminating tissues and structures ahead on the tool trajectory [2, 3].

Robotic surgery has demonstrated consistent results [4–6] for certain procedures in which these systems have found a niche. For many other procedures the initial cost, setup time, surgeon training overhead, and maintenance of a large system cannot be justified. If robotic surgery is to provide a benefit to a wider range of procedures then the robotic systems need to be smaller, of lower cost, and intuitive to use and require few additional resources to be applied into clinical practice. A number of hand guided robotic systems for surgery are emerging, for example, to assist in gripping tissues (laparoscopy), in guiding hand-held instruments, and in cutting applications (knee joint replacement surgery) [7–10]. Where feasible, the simplicity of hand guided robotic type instruments for surgery compared with the complexity of extensive manipulation robot systems is attractive in terms of the application of principles to a wide range of procedures at a reasonable cost. To accomplish this there is the need to engage more extensively with the less structured state of the working environment, as the point of registration is likely to be quite different to systems registered to scan data alone, for example. In some cases the reference may be the deforming tissue. For these devices, sensing systems, protocol, and configuration take on a new set of challenges.

In this paper the extension of an automated, arm supported robotic drill, used successfully in the operating room to produce precise cochleostomies, is explored as a hand guided unit. It relies on an innovative method for tactile sensing to determine the state of the tissue being drilled and tissue about to be drilled, enabling the surgeon to achieve consistent results.

Cochleostomy formation is a key step in cochlear electrode implantation. During this step the surgeon drills through the outer bone tissue of the cochlea and ideally onto, but not through, the underlying endosteal membrane. Following this step, debris is removed from the cochleostomy and electrode is inserted into the cochlea through a pool of antiseptic gel. If a surgeon penetrates the endosteum during the drilling process then residual hearing of the patient could be compromised. Preserving the endosteum in cochleostomy is regarded as ideal and difficult to achieve reliably. The arm supported drill has been designed to produce a consistent high quality hole without penetrating the underlying membrane. The innovative sensing scheme automatically discriminates the state of tissues during the cutting process and determines the presence of tissue interfaces and underlying structures ahead on the drilling trajectory. Using these unique properties it is able to avoid penetration of delicate interfaces and underlying tissues. As a hand guided tactile sensing device it is able to offer precise and consistent cutting of tissues, with some versatility of the trajectory during the surgical process.

## 2. Arm Supported Robotic Drill

The arm supported robotic drill was the first autonomous surgical robot deployed able to sense its own working environment as information rather than data values in order to discriminate states attributable to conditions of the cutting process in real-time and to use this information to control progress in flexible tissues. The drilling of a cochleostomy occurs on a single axis, and the recognition of prominent states enables the automatic selection of actuation strategy to expose the correct diameter of window onto the endosteum without penetrating the membrane. This is achieved in real-time.

**Figure 1:**
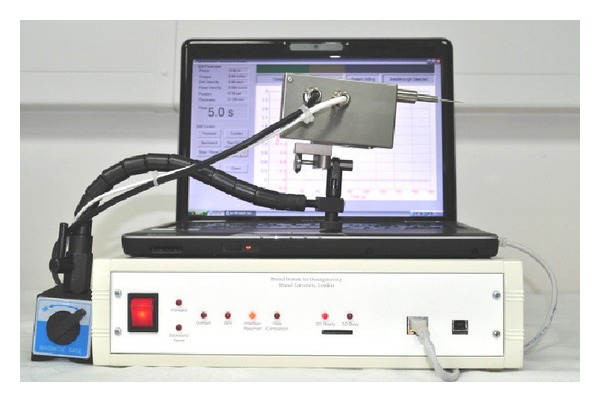
The surgical robot drilling system used in cochleostomy supported on a fixed flexilock arm.

The system consists of linear and rotational drives to feed and rotate standard surgical burrs. Currently the drill unit is attached to a flex-lock arm, permitting free movement to align the drill on the desired trajectory and then stabilization of the drill when drilling (Figure 1). Sensing through a discriminatory process of coupled features, feed force and torque transients enable perception of the critical phenomena of the tool working environment. Anticipation of conditions ahead of the tool before they are encountered enables discrimination of the approach to the critical endosteal membrane interface before it is reached. The drilling robot is able to autonomously adjust motion strategy with respect to the deforming tissues and achieve a consistent state in the result [11, 12].

## 3. Drilling Process

**Figure 2:**
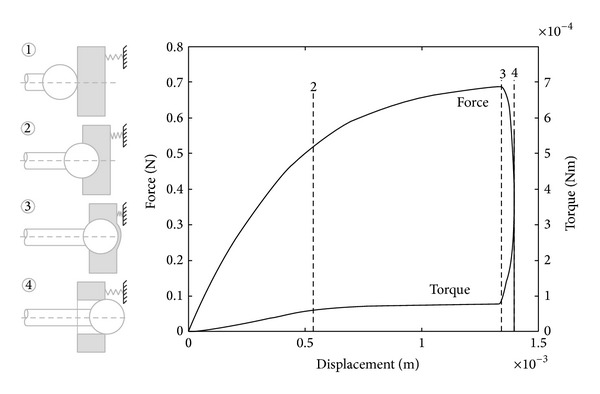
Simulated coupled drilling feed force and torque (assuming drilling through in cochleostomy) showing principal characteristics [12].

The mathematical model, reported in [12], predicts results shown in Figure 2 that help to describe typical features used by the tactile sensing scheme to identify the approach to a tissue interface such that penetration can be avoided. The drill bit feed force and torque are plotted as functions of displacement. The characteristics indicate clear changes in transients between coupled signals that correspond to stages in the process. In this simulation feed rate is assumed to be constant. The force and torque transients clearly show the point at which hole depth is equal to the burrs radius at stage 2 at approximately 0.5 mm and is indicated by an observable change in gradient of the torque transient. Onset of breakthrough occurs at stage 3 at approximately 1.3 mm resulting in the sharp increase and subsequent roll-off in the force signal. Amongst other properties and tissue behaviour, these coupled features of the sensory transients are used to anticipate the position of the tissue interface precisely. If drilling did not cease at this point then the hole would be completed at stage 4, at approximately 1.4 mm. The force and torque would then fall to zero when full penetration occurred. If penetration is allowed to take place, then in reality the tip of the drill bit will have penetrated much further beyond the tissue interface than is necessary to complete the removal of bone tissue of the cochlea as the tissues are flexible and will have deflected significantly in response to tool forces prior to penetration. Avoiding penetration is important in the process to minimise trauma of the hearing organ, as is the amplitude of disturbances induced during the drilling process [13].

When drilling in practice the force transients are affected by many disturbances and are not as clear as indicated in Figure 2. In reality tissue inconsistency, debris, involuntary disturbances of the patient, and other disturbances are present. By using the automated discriminatory approach above, the system is able to identify the approaching condition of interface penetration before it occurs.

The process of sensing and robot control is entirely through a hardwired control unit with the surgeon retaining executive control. Autonomous perception of critical phenomena and structures is completed using the coupled force and torque drilling transients, described above, in real-time. The automated selection of control strategies enables a precise and consistent result with respect to the flexible tissues to be achieved. The most important objective of the system is to prepare the window on the endosteum.

**Figure 3:**
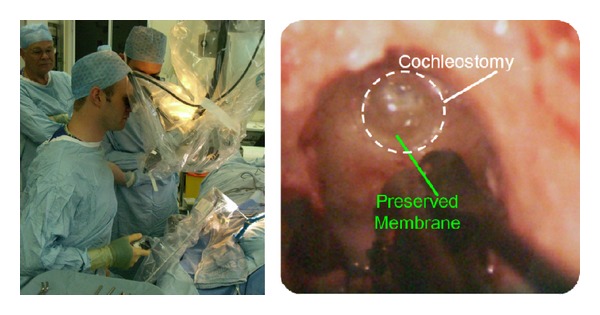
The arm supported drill used in operating room preparing a cochleostomy [12].

In Figure 3, the surgeon is holding the handset that enables executive control of fine alignment and on-off control of the autonomous process in operating room. Feedback is by observation of behaviour under the binocular microscope [12, 13]. Standard surgical drilling burrs are used. On completion of the drilling process, the surgical robot is removed. As described earlier, the cochlear electrode is then fed through a droplet of antiseptic gel placed within the cochleostomy. The smooth prepared access enables smooth insertion of the electrode.

## 4. Disturbances

The innovative sensing method used is well proved for discriminating tissue types, tissue structures, and tissue behaviour in the drilling process. Exposure to different disturbances has shown that involuntary patient disturbances are automatically classified as different types, as are knocking and a variety of manual types of disturbance while drilling from a flex-lock arm. The automated drilling process and ability for sensing have been shown to be unaffected on drilling trajectories up to 45 degrees from the perpendicular to a tissue interface [14].

**Figure 4:**
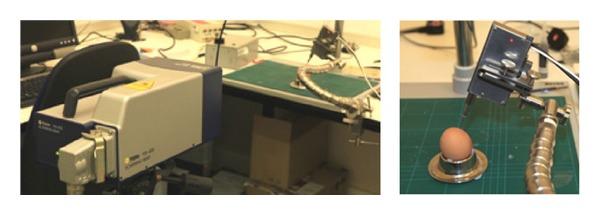
Setup of a laser Doppler vibrometer to evaluate disturbance velocity amplitude [14].

During trials for robustness with respect to forced disturbance [14] the drilling system was exposed to impact disturbance to the support arm applied to different axes when drilling eggshells and porcine cochlea. A laser Doppler vibrometer was used to obtain noncontact evaluation of disturbance velocity amplitude.  Figure 4 shows the experimental setup. Disturbances by controlled knocking at the support arm from different directions and supporting table were introduced to simulate inadvertent physical disturbance with the drilling system in the operating room. The successful results showed automatic discrimination of disturbance type, whether patient/operator or tissue induced, and led to the appropriate automatic control action toward completing or aborting the process. As would be expected a certain degree of compliance is helpful to the process.

**Figure 5:**
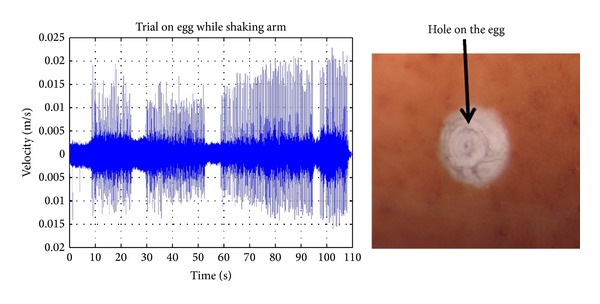
Completed hole and corresponding disturbance velocity transients [14].

Figure 5 shows an example of a completed hole and the corresponding disturbance velocity transients applied to the arm. Peak amplitude is 20 mm/s. The corresponding hole shown in Figure 5 is through the shell of a raw egg, a phantom for the cochlea, which is typical of many trials [10]. The figure shows that the tissue of the shell has been removed to expose a window onto the membrane of a diameter required for electrode insertion. The process of controlled knocking has not confused the system and the task has been accomplished without being disturbed. This shows that the sensing scheme of the drill offers robustness to environmental disturbances. The tolerance to disturbances also suggests tolerance to a variety of operator disturbances when guided by hand. The results from the current investigation on operator disturbance levels are not complete at this time and will affect the design of the drill unit.

## 5. Hand Guided Drill

There are many drilling tasks in surgery where flexibility in the drilling trajectory is needed during the process. A good practical example is when drilling into a convex hard surface, as is the case when drilling into the basal turn of the cochlea. Initial cutting without slip is achieved more readily when the drilling trajectory is normal to the surface. When initial cutting has been achieved, the drill can be orientated onto the desired trajectory toward the scala. A surgeon can identify this trajectory through exposed anatomical features following a posterior tympanotomy.

**Figure 6:**
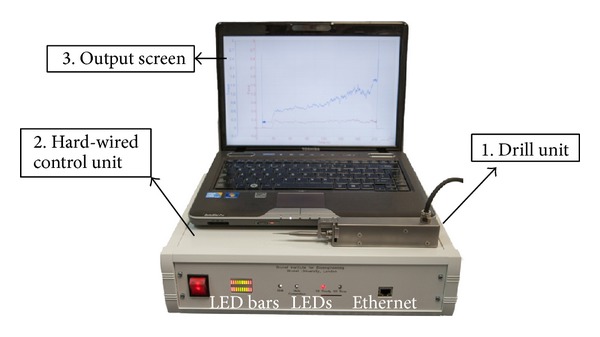
The experimental hand guided surgical robot drill system.

Similar to the arm supported system described earlier, the hand guided system consists of (1) a drilling unit, (2) a hard-wired unit for interpreting sensory signals and drill drive control, and (3) a PC screen for operator visual feedback. The system elements are shown in Figure 6.

**Figure 7:**
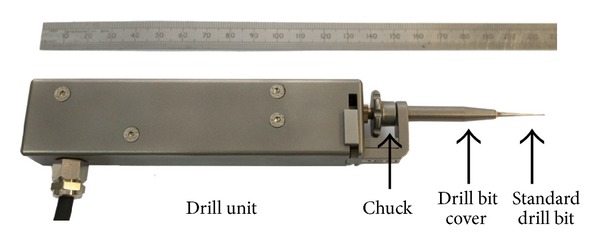
The hand guided robotic drill unit.

The drill unit comprises drill bit rotation drive and sensing elements and is shown in Figure 7. Standard drill bits are readily changed using the chuck. Feed force is measured by a displacement sensor and torque is measured using drive current of the DC motor. The control unit has a two-tier hierarchy: servo level and high-level controllers. The servo controls the rotation drive of the drill at 40 Hz and communicates with the PC through ethernet connection. The high-level controller responds to key stages and states of the drilling process by selecting predefined strategies. The selection is based on the interpreted state of the drilling process where the high-level controller discriminates characteristics in the coupled sensory transients indicating the onset of breakthrough. All control system and sensory functions operate in hardware. Progress of the procedure is relayed to the clinician on the screen. The drilling process is also indicated by the LEDs on the control unit. The drill will stop rotating when the cochleostomy is complete. The LED bars on the control unit indicate contacting force level between the drill bit and tissue and provide indication to the operator on the most suitable feed force range for the task. This arrangement for the bars works well in experimental trials and demonstrates a useful principle for practice.

## 6. Results

Results presented here relate to laboratory trials on phantoms. The purpose was to assess the feasibility and performance of guiding such a robotic device by hand. The shell of raw eggs and porcine cochlea were used to present appropriate media and tissue interfaces. In all experimental investigations 1 mm diameter diamond burrs were used. The trials were first carried out on eggshells where there is similarity to the structure of a cochlea. Porcine cochleae have similar properties to human cochlea [15]. These were used in trials to demonstrate the production of cochleostomies with intact endosteum.

**Figure 8:**
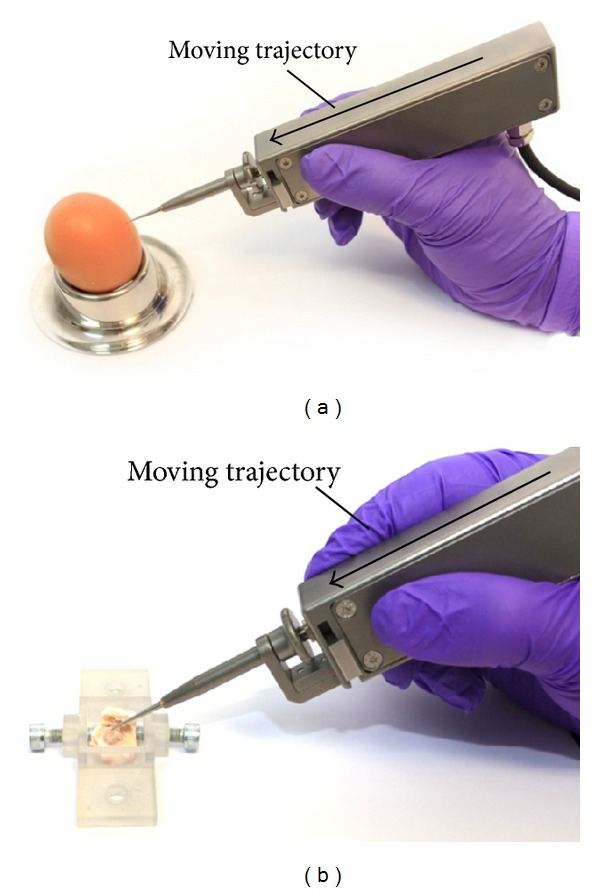
Drilling configurations with the drill unit held by the operator on raw eggshell and porcine cochlea.

**Figure 9:**
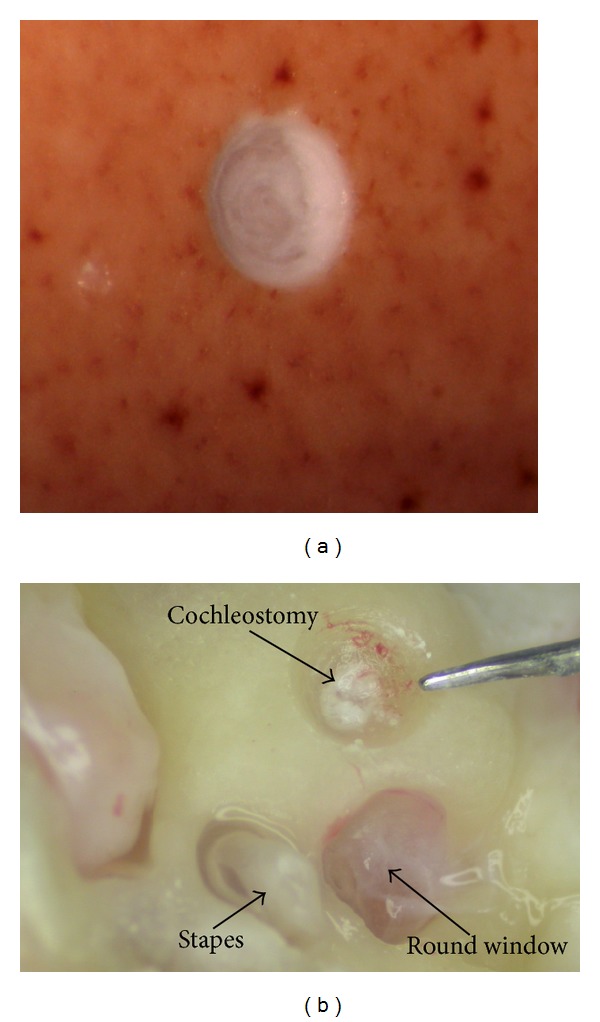
Intact underlying membranes following drilling through bone shell tissue of a raw egg and a porcine cochlea, respectively.

In Figure 8 the experimental configuration is shown. During these trials, the drill unit was gripped in the hand of the operator between thumb and forefinger with the hand providing support by resting on the bench. The arrow indicates the trajectory of motion imparted on the drill by the operator. Typical drilling results are presented in Figure 9 for both raw eggshell and porcine cochlea. In each case the underlying membrane remained successfully intact.

**Figure 10:**
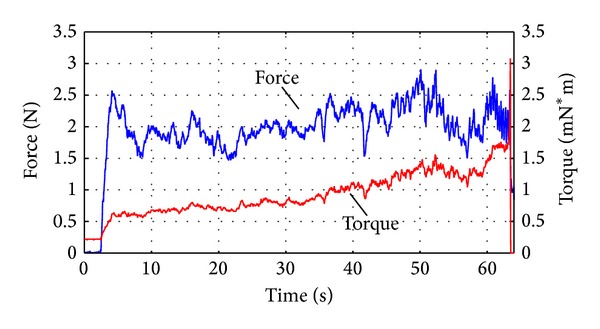
Typical coupled force and torque transients of the hand guided drill.

Typical coupled force and torque transients for drilling a porcine cochlea are shown in Figure 10. Usual sensory characteristics are present for contact, force building, and completion to the interface. The force level during drilling has a mean value of 1.99 N over the range from 1.4 N to 2.86 N. The operator begins by increasing feed force to ensure that the drill is cutting and is stable on the surface. The result is an initial force building transient. Following this period, the fluctuating force amplitude is primarily due to unsteady motion imparted by the operator.

**Figure 11:**
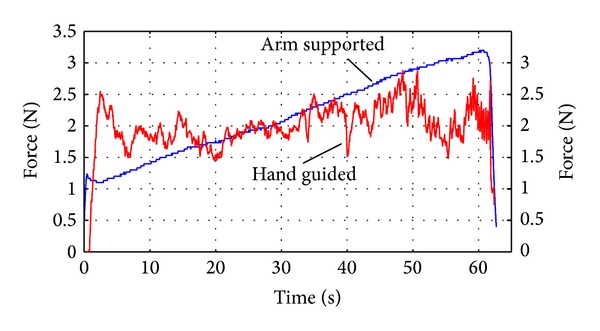
Contrasting force transients between the hand guided and automatically actuated drill in the laboratory.

Figure 11 provides contrast between reactive forces transients of hand guided and automated arm supported drill when drilling in the laboratory. As would be expected, the amplitude of disturbances is significantly greater for the hand guided system as opposed to the arm supported system since the stiffness of the drill unit in the feed direction is similar and the system is subject to involuntary operator disturbances. In the real operating environment both systems will be subjected to patient disturbances of similar and even greater disturbance amplitude [12]. The figure shows that peak feed force values are similar. There is a difference in operation between the two systems; the arm supported drill begins with a lower peak whereas the initial force peak under operator guidance is greater to reinforce stability. The hand guided system guides the operator toward a constant value of feed force whereas the automatic system increases over the period shown. The feed force is limited when using the automatic arm supported drill; however in the test result shown the force limit had not been reached. These results indicate the need to adjust compliance for the hand guided system and to achieve the compromise that will attenuate operator induced disturbances while maintaining stability.

## 7. Conclusion

This paper describes an investigation to contrast automated drilling by a surgical robot, supported by a fixed arm, with a robotic device that is hand guided. In each case the advanced discriminatory sensing scheme was used to control the state of the drilling process for cochleostomy formation in the laboratory. These trials had the aim of demonstrating preservation of the underlying endosteum while bone tissue is removed. Raw eggshells and porcine cochlea were phantoms used. These enabled investigation of the penetration of hard bone shell tissue and the physical verification of the intact membrane on hole completion.

The hand guided tool has advantage of versatility of the drilling trajectory and the tolerance to initiate cutting on a hard convex surface. The automated system imposes less force and disturbance level; however both systems can achieve the same ideal results. In these early results, the compliance was insufficient to reduce disturbances imparted by the operator when compared to the arm supported drill. Experimentation with the means to minimise reactive force disturbance is a subject of current investigation as the sensing approach and tolerance of the system to disturbances in the delicate procedure of cochleostomy offer feasibility of advantage in practice.

## References

[1] Cao T, Li X, Gao Z, Feng G, Shen P (2010). Automatic identification of otological drilling faults: an intelligent recognition algorithm. *The International Journal of Medical Robotics and Computer Assisted Surgery*.

[2] James C, Albegger K, Battmer R (2005). Preservation of residual hearing with cochlear implantation: how and why. *Acta Oto-Laryngologica*.

[3] Zou J, Bretlau P, Pyykkö I, Starck J, Toppila E (2001). Sensorineural hearing loss after vibration: an animal model for evaluating prevention and treatment of inner ear hearing loss. *Acta Otolaryngologica*.

[4] Guthart GS, Salisbury KJ Intuitive telesurgery system: overview and application.

[5] Jakopec M, Rodriguez y Baena F, Harris SJ, Gomes P, Cobb J, Davies BL (2003). The hands-on orthopaedic robot “acrobot”: early clinical trials of total knee replacement surgery. *IEEE Transactions on Robotics and Automation*.

[6] Lonner JH, John TK, Conditt MA (2010). Robotic arm-assisted UKA improves tibial component alignment: a pilot study. *Clinical Orthopaedics and Related Research*.

[7] Lonner JH, Kerr GJ (2012). Robotically assisted unicompartmental knee arthroplasty. *Operative Techniques in Orthopaedics*.

[8] Jaramaz A, Nikou C, Simone A (2013). Naviopfs for unicondylar knee replacement: early cadaver validation. *The Bone & Joint Journal B*.

[9] Schuller B, Rigoll G, Can S, Feussner H Emotion sensitive speech control for human-robot interaction in minimal invasive surgery.

[10] Nelson CA, Zhang X, Shah BC, Goede MR, Oleynikov D (2010). Multipurpose surgical robot as a laparoscope assistant. *Surgical Endoscopy*.

[11] Brett PN, Taylor RP, Proops D, Griffiths MV, Coulson C An autonomous surgical robot applied in practice.

[12] Taylor R, Du X, Proops D, Reid A, Coulson C, Brett PN (2010). A sensory-guided surgical micro-drill. *Proceedings of the Institution of Mechanical Engineers C: Journal of Mechanical Engineering Science*.

[13] Coulson CJ, Zoka Assadi M, Taylor RP (2013). Smart micro-drill for cochleostomy formation: a comparison of cochlear disturbances with manual drilling and a human trial. *Cochlear Implants International*.

[14] Du X, Assadi MZ, Jowitt F (2013). Robustness analysis of a smart surgical drill for cochleostomy. *The International Journal of Medical Robotics and Computer Assisted Surgery*.

[15] Pracy JP, White A, Mustafa Y, Smith D, Perry ME (1998). The comparative anatomy of the pig middle ear cavity: a model for middle ear inflammation in the human?. *Journal of Anatomy*.

